# Supplementary material for: VIP interneurons in mouse primary visual cortex selectively enhance responses to weak but specific stimuli
Source: eLife. 2020 Oct 27;9:e55130. doi: 10.7554/eLife.55130 (PMC7591255; doi:10.7554/eLife.55130)
Supplement: Figure 1—source data 1. [file elife-55130-fig1-data1.docx]

|  | **Cells** | **Sessions** | **Mice** |
| --- | --- | --- | --- |
| **Cux2** | 338 | 7 | 4 |
| **Rorb** | 640 | 4 | 3 |
| **Rbp4** | 91 | 4 | 3 |
| **Ntsr1** | 297 | 6 | 4 |
| **Sst** | 66 | 8 | 5 |
| **Vip** | 63 | 6 | 3 |
