# Supplementary material for: VIP interneurons in mouse primary visual cortex selectively enhance responses to weak but specific stimuli
Source: eLife. 2020 Oct 27;9:e55130. doi: 10.7554/eLife.55130 (PMC7591255; doi:10.7554/eLife.55130)
Supplement: Transparent reporting form [file elife-55130-transrepform.pdf]

eLIFE
1st Floor
24 Hills Road
Cambridge CB2 1JP, UK
P 01223 855340
W elifesciences.org
T @elife

eLife's transparent reporting form

We encourage authors to provide detailed information *within their submission* to facilitate the interpretation and replication of experiments. Authors can upload supporting documentation to indicate the use of appropriate reporting guidelines for health-related research (see EQUATOR Network), life science research (see the BioSharing Information Resource), or the ARRIVE guidelines for reporting work involving animal research. Where applicable, authors should refer to any relevant reporting standards documents in this form.

If you have any questions, please consult our Journal Policies and/or contact us: editorial@elifesciences.org.

Sample-size estimation

- You should state whether an appropriate sample size was computed when the study was being designed
- You should state the statistical method of sample size computation and any required assumptions
- If no explicit power analysis was used, you should describe how you decided what sample (replicate) size (number) to use

Please outline where this information can be found within the submission (e.g., sections or figure legends), or explain why this information doesn't apply to your submission:

As stated in the Methods section under Experimental Animals: "Sample size was determined qualitatively to balance repeated experiments for each layer/Cre-line and to preserve the breadth of survey."

Replicates

- You should report how often each experiment was performed
- You should include a definition of biological versus technical replication
- The data obtained should be provided and sufficient information should be provided to indicate the number of independent biological and/or technical replicates
- If you encountered any outliers, you should describe how these were handled
- Criteria for exclusion/inclusion of data should be clearly stated
- High-throughput sequence data should be uploaded before submission, with a private link for reviewers provided (these are available from both GEO and ArrayExpress)

Please outline where this information can be found within the submission (e.g., sections or figure legends), or explain why this information doesn't apply to your submission:

eLife Sciences Publications, Ltd is a limited liability non-profit non-stock corporation incorporated in the State of Delaware, USA, with company number 5030732, and is registered in the UK with company number FC030576 and branch number BR015634 at the address 1st Floor, 24 Hills Road, Cambridge CB2 1JP | August 2014

Supplementary Table 1 contains the number of unique mice and imaging sessions for each Cre line.

The Methods section, under *Visual Stimulus*, describes that each visual stimulus condition was repeated 15 times per experiment, except for the 0% contrast (blank) condition which was repeated 30 times per experiment.

The Methods section, under *Two photon imaging platform and image processing*, describes that "Mice were excluded for evidence of epileptiform activity, and individual imaging sessions were failed if there were signs of bleaching, saturation, excessive z-drift, or animal stress, among other factors."

Statistical reporting

- Statistical analysis methods should be described and justified
- Raw data should be presented in figures whenever informative to do so (typically when N per group is less than 10)
- For each experiment, you should identify the statistical tests used, exact values of N, definitions of center, methods of multiple test correction, and dispersion and precision measures (e.g., mean, median, SD, SEM, confidence intervals; and, for the major substantive results, a measure of effect size (e.g., Pearson's r, Cohen's d)
- Report exact p-values wherever possible alongside the summary statistics and 95% confidence intervals. These should be reported for all key questions and not only when the p-value is less than 0.05.

Please outline where this information can be found within the submission (e.g., sections or figure legends), or explain why this information doesn't apply to your submission:

All statistical analyses are described in the Methods section under the *Analysis*.

As stated in the second paragraph of the Main Text, and described in detail in the Methods section, a bootstrapped Chi-squared test was used to determine whether each neuron responded to the visual stimulus set and an inclusion criteria for further analysis of responsive neurons was set at $p<0.01$.

As stated in the legends for Figures 1a and 2, all tuning curves are shown as mean +/- SEM.

As stated in the legend for Figure 1c, vector sums are compared against the 90% confidence interval for a null distribution with uniform direction selectivity (shown as a gray-shaded region).

As stated in the legend for Figure 1e, and described in detail in the Methods section under *Response Significance by Stimulus Condition and test for suppression by contrast*, the test for suppression by contrast through a bootstrap analysis with a criteria for significance set at a family-wise type-1 error rate of $p<0.05$.

(For large datasets, or papers with a very large number of statistical tests, you may upload a single table file with tests, Ns, etc., with reference to sections in the manuscript.)

Group allocation

- Indicate how samples were allocated into experimental groups (in the case of clinical studies, please specify allocation to treatment method); if randomization was used, please also state if restricted randomization was applied
- Indicate if masking was used during group allocation, data collection and/or data analysis

Please outline where this information can be found within the submission (e.g., sections or figure legends), or explain why this information doesn't apply to your submission:

This study only consisted of a single experimental group.

Additional data files ("source data")

eLIFE
1st Floor
24 Hills Road
Cambridge CB2 1JP, UK
P 01223 855340
W elifesciences.org
T @elife

- We encourage you to upload relevant additional data files, such as numerical data that are represented as a graph in a figure, or as a summary table
- Where provided, these should be in the most useful format, and they can be uploaded as "Source data" files linked to a main figure or table
- Include model definition files including the full list of parameters used
- Include code used for data analysis (e.g., R, MatLab)
- Avoid stating that data files are "available upon request"

Please indicate the figures or tables for which source data files have been provided:C

Data analysis code is available at https://github.com/everythingevolves/Contrast_Analysis. Stimulus, calcium imaging, and locomotion data will be shared upon publication via Figshare or Datajoint.

eLife Sciences Publications, Ltd is a limited liability non-profit non-stock corporation incorporated in the State of Delaware, USA, with company number 5030732, and is registered in the UK with company number FC030576 and branch number BR015634 at the address 1st Floor, 24 Hills Road, Cambridge CB2 1JP | August 2014
